# Combination of measures of handgrip strength and red cell distribution width can predict in-hospital complications better than the ASA grade after hip fracture surgery in the elderly

**DOI:** 10.1186/s12891-017-1738-3

**Published:** 2017-08-30

**Authors:** Hyung-Min Ji, Jun Han, Hi-Won Bae, Ye-Yeon Won

**Affiliations:** 1 Department of Joint Surgery, Siheung 21C Hospital, Siheung, South Korea; 2 Department of Orthopedics, Ajou University School of Medicine, Suwon, South Korea

**Keywords:** Erythrocyte indices, Hand strength, Proximal femur, Hip fractures, Comorbidity, Prediction

## Abstract

**Background:**

Early detection of a high-risk patient following hip fracture surgery is of paramount clinical importance. American Society of Anesthesiologists (ASA) grading is an easy and efficient index in predicting a worse outcome. The red cell distribution width (RDW) and handgrip strength, are gaining interest as a prediction tool as well. Accordingly, the objective of this study was to investigate the potential association between ASA, RDW and grip strength and detect the effects of combining RDW and grip strength for predicting early complication after hip fracture surgery in the elderly.

**Methods:**

Eighty-three consecutive patients operated with hip fracture surgeries were identified retrospectively. Age, gender, diagnosis, RDW, handgrip strength and ASA grade were recorded. Admission to the intensive care unit (ICU), length of ICU stay, transfer to other departments, in-hospital death, and readmission were investigated as early complications. Logistic regression analysis was applied to evaluate the estimates in predicting complications, and receiver operating characteristics curves were constructed to compare the estimates and decide which method is more accurate.

**Results:**

After the surgery, 52% of the patients were admitted to the ICU. From the analyses, RDW and grip strength had no significant relation with each other. However, the ICU stay was correlated with RDW and grip strength but not for the ASA grade. A higher ASA grade and grip strength could independently predict ICU admission. The combination of RDW with grip strength outweighed the ASA grade in predictive ability.

**Conclusions:**

The current study indicated that combining RDW and grip strength measures can be efficient and clinically relevant in predicting early postoperative complications after fragility hip fracture in the elderly. Due to the objectivity and availability of those two approaches, patient care, and functional outcomes are expected to be improved by adopting these measures in the clinical setting.

## Background

The aging population has to face the prospect of increased risk from hip fractures as they pose a significant health burden for the elderly worldwide [1]. The number of the elderly is continuously increasing in the United States [2], with the number of men and women above the age of 65 expected to reach 89 million by year 2050 in that country. In Korea, the total number of hip fractures is also estimated to increase 1.4-fold by 2025 from the current numbers in 2016 [3]. In the same report, the standardized mortality ratios for hip fracture have been higher than those in the general population for all age groups [3].

Surgical intervention is a definite treatment of choice for most hip fractures to relieve pain and facilitate early mobilization. The 30-day mortality rate after hip fracture surgery has declined in the last decade and has been reported to range from 1.4 to 12.1% and in a recent report, but has reached a plateau [1]. The timing of the return to optimal function is determined by preoperative comorbidities and perioperative complications [4, 5]. Prediction of complications after hip fracture surgery in elderly subjects is of crucial importance in preventing adverse effects, as the patients are still vulnerable after the surgery. Identifying subjects with risk factors can prevent an unexpected adverse event, and every medical resource such as with a multi-disciplinary team approach should be focused on this purpose, as already shown to significantly reduce the 1-year mortality [6]. Pre-surgical evaluation tools such as the Charlson comorbidity index [7], the Nottingham Hip Fracture Score [8], and the orthopedic version of the Physiological and Operative Severity Score for the Enumeration of Mortality and Morbidity [9] have been put together and provide accurate risk prediction and help in decision making and clinical planning. The American Society of Anesthesiologists (ASA) scoring system was utilized for estimating the patient’s physical suitability for surgery [10] and has been reported to be a strong predictor of complications related to the hip fracture operation and mortality [4, 6, 11–13]. The system is also relatively easy to use, and there is considerable agreement strength for the reliability of the system in the orthopedic trauma patients [14]. However, it is also true that the ASA scoring system’s reliability has been questioned among anesthesiologists from differing backgrounds [14].

An additional predictive index to consider is the red cell distribution width (RDW). RDW is a commonly applied laboratory parameter reflecting the heterogeneity of circulating erythrocytes and has traditionally been used for differential diagnosis of anemia [15]. However, numerous studies have shown that increased RDW can predict negative outcomes after disease conditions [15] including mortality after a hip fracture surgery [16]. Another index to consider is the handgrip strength, as it is also a strong surrogate measure of overall muscular strength [17], and a weak handgrip strength is an independent predictor of complications and mortality after surgical procedures [18]. Increased handgrip strength independently predicted fairer functional recovery in women sustaining hip fracture surgery [19] and shortened hospital stay in arthroplasty patients [20]. Therefore, a combination of these two readily available parameters (RDW and handgrip strength) would provide a reference for early risk prediction for hip fracture surgery. Yet, to the best of our knowledge, no detailed study on the predictive performance of the combination of these two indices for the above indication has been published.

The authors hypothesized that the combination of RDW and handgrip strength would provide a comparable prediction of early complication as the ASA grade after hip fracture surgery in the elderly. The objective of the study was therefore to examine the relationship between RDW, perioperative handgrip strength, ASA grade, and early complications such as admission to the intensive care unit, transfer to the other department, in-hospital mortality, and readmission within 3 months after discharge. Another objective for the study was to compare the acuity of the prediction between the combination and the ASA grade and to decide which is a better predictive tool. The results of this study would aid in the evaluation of the hip fracture patient and decision-making.

## Methods

The authors conducted a power analysis for multiple logistic regression as described in the literature [21] for a retrospective cross-sectional study. The rate of “admission to the intensive care unit (ICU)” was assumed to be 30% in hip fracture patients. Clinically relevant difference in the complication rate was supposed to be 10% [4]. The analysis was completed with a desired one-sided alpha of 0.05 and a power of 0.80. The variance inflation factor was assumed to be 10%. With these characteristics, the patient size required was 83, which coincided with the actual number of patients enrolled in the current study.

Elderly patients older than 60 years who suffered a fragility hip fracture and received surgical intervention in a single institution were included in the current study. After obtaining approval by institutional review board in our institution, 83 consecutive patients (67 men and women) were enrolled. Via a retrospective chart review, the patients had experienced displaced femoral neck or intertrochanteric fracture and had received a surgical procedure at a single institution with a level I trauma center from November 2014 to February 2016. Patients older than 60 years old with fragility fractures caused by a low energy trauma were included in the study. However, 7 patients were younger than 60 years, although they had been suspected to suffer from low energy trauma. One hundred seven patients with 107 hips were operated during this period. Patients who also had sustained poly-trauma or injury in another part of the body such as the head or spine were excluded (10) leaving 100 hips. Subjects who failed to properly follow the instructions for the initial evaluation of grip strength due to severe dementia or cognitive dysfunction were also excluded from the study (5). Two more patients were also excluded because of having a previous metabolic bone disease or metastatic bone cancer. This left 83 patients as part of the study. Baseline clinical characteristics and demographics were collected from the patients. Age at the time of surgery, gender, diagnosis (femoral neck fracture or intertrochanteric fracture), RDW, handgrip strength, the ASA grade, admission to the ICU, duration of ICU care, transfer due to the postoperative complication, in-hospital mortality, and the length of hospital stay were recorded (Table 1). Among these variables, RDW and handgrip strength was measured prospectively.

**Table 1:** Baseline demographics and rates of early complications in patients with hip fractures

Variable	Total (*N* = 83)	Grade 1 (*N* = 27)	Grade 2 (*N* = 40)	Grade 3 (*N* = 15)	Grade 4 (*N* = 1)
Age (Year)^*^	79.5 (6.7)	78.3 (6.3)	79.0 (7.1)	81.3 (5.9)	69.0
Women (%)	80	74.1	88.1	73.3	100.0
Intertrochanteric fracture (%)	63.5	51.9	71.4	66.7	100.0
RDW (%)	14.3 (1.6)	13.8 (1.0)	14.3 (1.6)	14.8 (1.8)	19.6
Grip strength (kg)	37.0 (18.0)	40.2 (17.1)	30.4 (19.1)	29.8 (11.5)	18.8
ICU care (%)	52.9	44.4	47.6	80	100
Transfer out (%)	8.2	0	9.5	20	100
In-hospital mortality (%)	4.7	0	4.8	13.3	100
Readmission (%)	5.9	3.7	7.1	6.7	100

^*^The values are given as the mean with the SD in parentheses

Total and divided in accordance with their ASA grade

The first RDW was part of the routine complete blood count battery of results (Samsung LABGEOHC10 Hematology Analyzer, Samsung Electronics Co., Suwon, Korea) included on the day of admission as part of the analysis before any alteration of RDW due to a hematologic intervention like an allogeneic blood transfusion could occur. Within 6 h after the hospitalization, isometric maximal handgrip strength was measured with a digital hydraulic dynamometer (Jamar Plus, Sammons Preston Rolyan, Bolingbrook, IL, USA) by an orthopedic resident in charge of the ER patients. The assessment of grip strength using a handheld dynamometer has proven to be credible and valid in hospitalized elderly patients [22]. After sufficient pain control, the patients were asked to be in the upright position as much as possible and tolerable. The shoulder was adducted and neutrally rotated, and the elbow was flexed at 90° with the forearm and wrist in a neutral position as recommended [23]. The handgrip hand was adjusted 4.8 cm apart from the main frame which was reported to be the optimal handgrip position for the dynamometer model [24]. The patient was requested to use his or her dominant or their best hand. The best performance was recorded from 3 attempts of maximal voluntary contraction, which was performed at 30-s intervals. The strength was measured in kilograms, with a precision of 0.1 kg. All patients were initially admitted to the orthopedic department at the authors’ institution until discharge. Most of the patients were operated within 48 h after admission. For some patients, surgery was delayed in order to receive medical clearance. The surgery was also delayed if the physician in charge decided that the correction of a medical illness would improve the patient’s condition and would outweigh the risk of a surgery delay and subsequent increased morbidity. All patients were assigned an ASA grade the night before the surgery by a resident from anesthesiology as part of their preoperative evaluation. The ASA grade was confirmed by the attending anesthesiologist at the morning before the surgery.

Generally, the ASA grade is determined as a five-level system. A higher ASA grade indicates a more morbid patient. A patient with a displaced femoral neck fracture was treated with either bipolar hemiarthroplasty or total hip arthroplasty. Primary osteosynthesis was tried in patients with little or no displacement or an intertrochanteric fracture. Following the fracture surgery, a patient was transferred to the ICU at the request of anesthesiologist and cared by the physician dedicated to the ICU and the anesthesiologist until the vital signs stabilized. The anesthesiologist confirmed the ASA grade but was not aware of the grip strength. The duration of ICU care was recorded. The duration was recorded as zero if there was no admission to the ICU. Transfer to another department was considered to address medically-related or non-orthopedic complications. Occasionally a transfer to the ICU from the general ward was considered. The patients were allowed to ambulate as soon as possible with a wheelchair, followed by walking aids, and to be discharged within 2 weeks of the operation. Patient death during the hospital stay was identified, and length of hospital stay was recorded. Readmission within 3 months after discharge was considered a complication as well as if the occasion of re-admission was related to the fracture and the surgery.

## Statistical analysis

The authors sought to determine if there was any relationship between the two risk evaluation tools (combination of RDW and grip strength, ASA) and decide which tool prevailed. A *p*-value <0.05 was considered statistically significant. Normality of data distribution was examined using the Kolmogorov–Smirnov test. Chi-square tests or Fisher exact tests were used accordingly to determine differences in the rates each complication between the groups. Independent t-test was used to establish differences in demographics. One-way ANOVA test with Tukey’s post hoc test was used for a comparison of variables such as the duration of ICU care, the rates of patient death during the hospital stay, length of hospital stay, readmission within 3 months after discharge amoung multiple groups (ASA 1, ASA 2, and ASA 3 / 4). Spearman or Pearson correlation coefficients were used for verifying relations between the ICU stay and RDW, grip strength. Univariate and multiple logistic regression analyses were carried out to compare the acuity in the prediction of postoperative complications between the two methods.

Receiver operating characteristics curve was constructed for each method. The area under curve (AUC) was calculated and compared between the two approaches. All data were analyzed using SPSS version 16.0 software (SPSS Inc., Chicago, IL, USA) and Medcalc statistical software (Medcalc version 16.8.4) (Medcalc Software bvba, Ostend, Belgium).

## Results

**Fig. 1:**
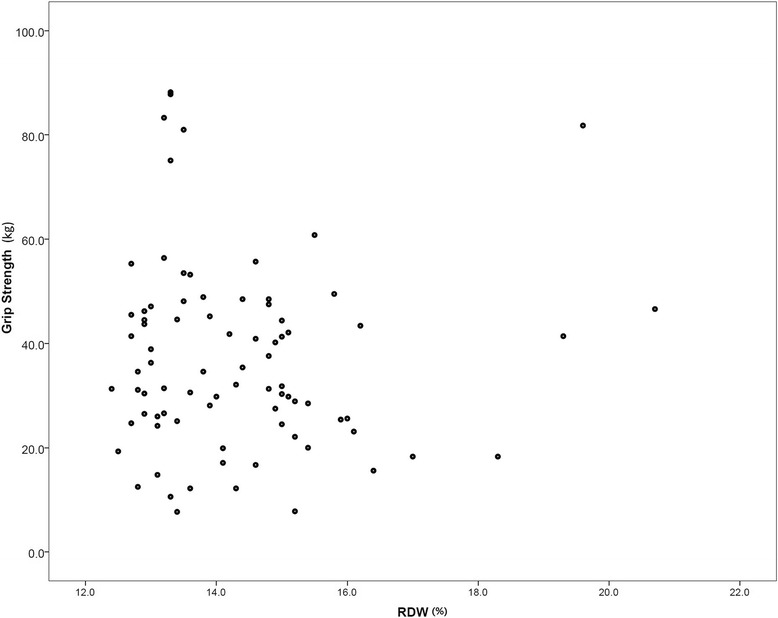
Scatterplot of red cell distribution width on x-axis and grip strength (kg) on y-axis

Patient demographics and hospital course results are presented in Table 1. There was no statistical difference for age among the groups classified with ASA grade. However, RDW and grip strength values were statistically different among the groups. There was no statistical difference in the frequency of women and intertrochanteric fracture among the groups. In addition, there was no difference in the rates of ICU admission, readmission, and in-hospital mortality among ASA groups while the rate of transfer out of orthopedic department was significantly different among the groups. There was no correlation between RDW and grip strength (Fig 1). The duration of ICU stay was positively correlated with RDW and grip strength (*r* = 0.303, *p* = 0.006; *r* = −0.290, *p* = 0.010 respectively) while there was no difference among the ASA groups.

**Fig. 2:**
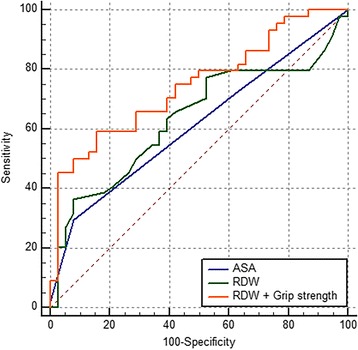
Receiver operating curves of American Society of Anesthesiologists (ASA) grade, red cell distribution width (RDW) alone, and combination of RDW and grip strength for predicting of intensive care unit admission

**Fig. 3:**
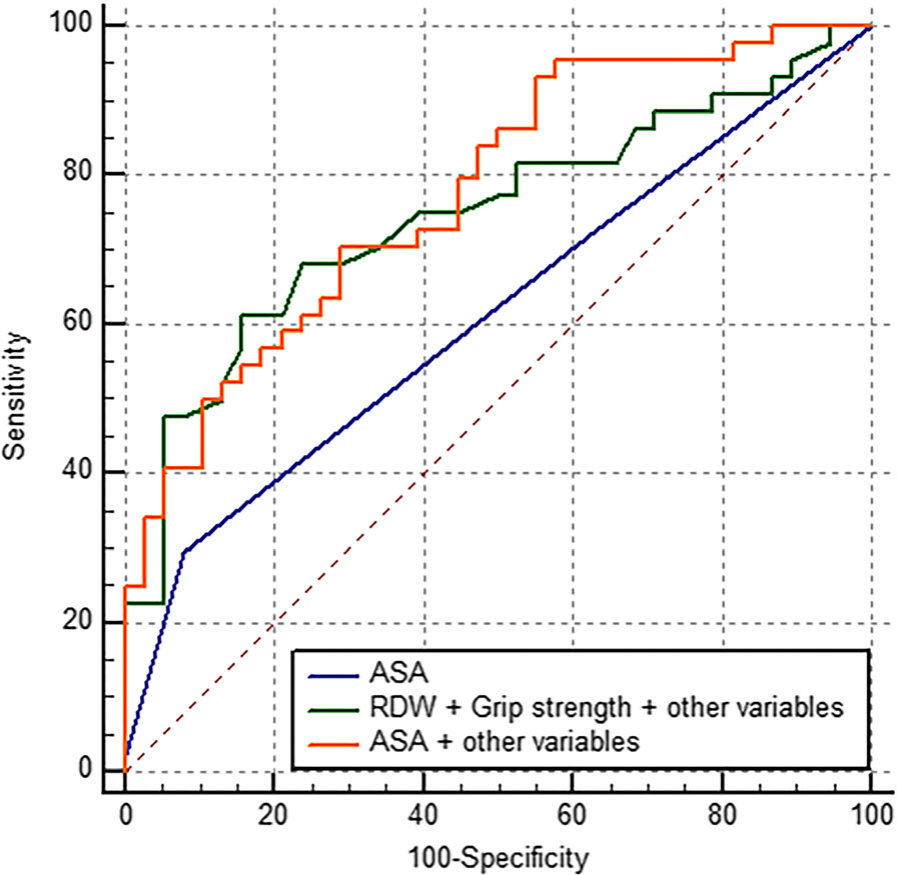
Receiver operating curves of American Society of Anesthesiologists (ASA) grade, combination of red cell distribution width (RDW), grip strength, and other variables, and combination of ASA grade and other variables for predicting intensive care unit admission

Univariate and multivariate logistic regression analysis displayed that the ASA grade alone and the grip strength alone could predict ICU admission (Table 2). Higher ASA grade, RDW increased the odds of ICU admission while grip strength decreased the odds. The comparison between the combination model and the ASA grade showed significance favoring the former (Fig. 2, Table 3, *p* = 0.048). When other variables were added to the combination model, it showed a significantly better prediction (Fig. 3, Table 3, *p* = 0.027). However, the differences disappeared when other variables were added to the ASA grade model. No model could predict the other complications such as transfer to the other department, in-hospital death, and readmission.

**Table 2:** Analysis of risk factor for intensive care unit admittance by univariate and multivariate logistic regression analysis

	R^2^ (%)		Odds ratio	95% confidence interval	*p* - value
ASA grade	2.7		1.99	1.06 to 3.75	0.033
RDW	6.4		1.36	0.98 to 1.90	0.044
Grip strength	19.8		0.95	0.92 to 0.98	0.002
Age	11.9		1.10	1.02 to 1.18	0.009
Gender	0.6		1.37	0.49 to 4.07	0.541
Diagnosis	5.2		2.33	0.92 to 5.94	0.076
ASA grade + other variables^*^	22.4	ASA grade	4.48	1.06 to 4.13	0.027
		Age	1.10	1.02 to 1.19	0.012
		Diagnosis	2.62	0.94 to 7.29	0.064
RDW + Grip strength	25.9	RDW	1.41	1.01 to 1.98	0.045
		Grip strength	0.95	0.92 to 0.98	0.001
RDW + Grip strength + other variables^*^	33.4	RDW	1.45	1.03 to 2.04	0.034
		Grip strength	0.93	0.90 to 0.97	0.001
		Age	1.04	0.96 to 1.13	0.329
		Diagnosis of FNF (vs. IF)	0.26	0.08 to 0.82	0.021

^*^Age, diagnosis

*ASA* American Society of Anesthesiologists, *RDW* red cell distribution width, *FNF* femur neck fracture, *IF* intertrochanteric fracture, *RDW* (%), Grip strength (kg)

**Table 3:** Comparison of area under curve (AUC) after a receiver operating characteristic analysis between prediction models

	AUC	95% confidence interval
ASA grade	0.61	0.50 to 0.72
Grip strength	0.73	0.63 to 0.83
RDW + Grip strength	0.75	0.64 to 0.84
ASA grade + other variables^*^	0.75	0.64 to 0.84
RDW + Grip strength + other variables^*^	0.78	0.67 to 0.86

^*^Age, diagnosis

## Discussion

To the best of our knowledge, this study is the first to show that the combination of RDW and grip strength measures can be substantially reliable in predicting postoperative outcome after fragility hip fracture in the elderly. The study also successively proved that this new approach is comparable in the credibility as with the ASA grade. Unlike the ASA grade, RDW and grip strength had a significant correlation with the duration of ICU stay. When RDW and grip strength were combined with age, gender, and diagnosis, the model showed fairer performance than the ASA grade did. Considering that RDW and grip strength is hardly influenced by the observer’s subjective opinion, the combination can help decision-making and preoperative planning in the clinical setting.

The results of our report are substantially supported by recent studies. Previous reports regarding the clinical significance of RDW and grip strength as a surrogate indicator of complications support the claims of the current study [4, 25, 26]. The ability to forecast long-term complication after hip fracture only by RDW have proved effective in one prospective cohort study [25]. Higher RDW predicted a higher mortality rate throughout the 4 years. One retrospective study examined 197 elderly patients with hip fracture and concluded that ASA grade was a strong predictor of the early complication [4]. Another prospective cohort study showed that the prediction of ASA grade might be augmented by evaluating RDW [26]. The study showed both ASA grade and RDW was an independent risk predictor of 2-year hip fracture mortality.

Grip strength is a popular measurement tool in the diagnosis of sarcopenia [17]. Sarcopenia and osteoporosis are closely related and may have a harmful synergistic effect and lead to the frailty syndrome, raising the mortality rate [13]. One prospective study showed handgrip strength assessment before beginning of rehabilitation after hip fracture surgery could independently predict the improved functional outcome in hip fracture women [19]. One recent study conducted in patients receiving elective hip and knee arthroplasty surgery proved that decreased grip strength could foretell prolonged hospital stay [20]. The authors theorized that lower grip strength might be associated with increased complications and reduced mobility.

This study is not free from limitations before its findings can be considered for application in a clinical setting. Foremost, as the study is a retrospective, it is possible that some complications or events were omitted or not thoroughly documented or treated. A likely omission could have changed the score of each measure in prediction of adverse events. Second, although the logistic regression was sufficiently powered to note for a complication rate of 10% difference in the whole cohort, there might be a possibility of insufficient power in discriminating the occurrence of other events by the evaluated models. The prevalence of ICU admittance was highest while other complications were relatively scarce. The difference of such complications could have been smaller than 10%, and this limitation warrants a further prospective study adequately powered to detect for minute differences. Third, ASA grade was considered as an interval variable not as an ordinal variable during regression analysis to compare AUC between two methods. Such consideration might have complicated the fair comparison. Finally, other possible confounding factors like delay to surgery, premorbid mental status, and medical illness were not included in the analysis. If these variables were included, the prediction ability of each tool might have been different. Patients with severe dementia were also excluded. Previous studies, however, have shown that RDW is closely correlated to the patient’s comorbidity and that it could be an independent risk factor regardless of the patient’s basic characteristics [15, 16]. Accurate prediction with complex scoring systems based on the patient’s comorbidity as shown in the literature might seem attractive [7, 9, 27]. However, obtaining such complex information and combining with complicated calculations may not always be plausible in an acute trauma setting. Therefore, prediction of complications by combining RDW with grip strength without obtaining a comprehensive past medical history is of clinical relevance.

Our study had several interesting findings. Duration of ICU care was related to both RDW and grip strength while the ASA grade wasn’t. This finding parallels another result that the combination of RDW and grip strength showed a better prediction than the ASA grade alone. ICU admission is one of the pitfalls that may lead to a worse outcome in the care of fragility hip fracture patients. Delirium in the ICU care is an extremely frequent complicating the results of surgery [28] leading to poorer function and increased mortality [29]. Minimizing the length of staying ICU is one of the main strategies in its prevention [30]. As one of the main purposes of the ASA grading should be a prediction of the ICU care and the length of stay there, a possible lack of prediction ability and reliability in the ASA grade would naturally render RDW and grip strength to be an essential predictive tool in the care of these patients.

In the study, RDW and grip strength was not correlated. This finding suggests these two surrogates might affect the outcome in its own way and complementing the other. RDW represents chronicity of the subject’s condition. A higher RDW may reflect a worsened medical condition and history of medical illness [15]. On the contrarily, lower grip strength might reflect the subclinical possibility of the condition leading to the frailty syndrome. It is possible that RDW might be increased while grip strength is preserved. Such a condition may indicate that the medical comorbidity hasn’t prevailed to the level of frailty yet. An exactly opposite situation is also possible. Preserved RDW might indicate a relatively stable medical condition while the decreased grip strength might suggest an increased vulnerability to the stressful situation, forecasting a future complication. Further study would be required to clarify such a sophisticated relationship between RDW and grip strength.

The current methodology could be readily used in the clinical setting and aid in the care of fragility hip fracture patients. The fractured hip itself is a striking marker of the collapse of well-being in that person. Preoperative function and postoperative complications may equally complicate recovery of function and have enormous medical and socio-economic consequences for the patient. The surgical treatments and anesthesia techniques, as well as those for pain control, has improved during last decade. However, the mortality rate and estimated survival for this group of patients are still disappointing [31]. Early identification of high-risk patients with a multidisciplinary approach has shown a favorable outcome and less complication after surgery [4]. As such, a vigorous effort is needed to develop a more accurate system that can in advance identify patients at risk. The findings of the current study can be utilized and further be improved through additional studies in contributing to the fight against the fragility hip fracture.

## Conclusion

The ASA grade, as well as a combination of RDW with grip strength, were markedly successful in predicting ICU admittance, which is one of the grave early complications following fragility hip fracture surgery. The predictability of both methods was comparable while the latter prevailed with statistical significance. Both RDW and grip strength was correlated with length of ICU stay while the ASA grade didn’t show such trend. Prediction by the combination was improved with a simple knowledge of the diagnosis of the fracture, and the prediction was significantly more accurate than the ASA grade alone. As RDW and grip strength measurement is both amicable in the clinical setting, the combination of these two would facilitate early detection of the high-risk individual and improve the outcome after hip fracture surgery.

## Abbreviations

ASA: American society of anesthesiologists
AUC: Area under curve
ICU: Intensive care unit
RDW: Red cell distribution width
